# Colonization factors of *Campylobacter jejuni *in the chicken gut

**DOI:** 10.1186/1297-9716-42-82

**Published:** 2011-06-29

**Authors:** David Hermans, Kim Van Deun, An Martel, Filip Van Immerseel, Winy Messens, Marc Heyndrickx, Freddy Haesebrouck, Frank Pasmans

**Affiliations:** 1 Department of Pathology, Bacteriology and Avian Diseases, Faculty of Veterinary Medicine, Ghent University, Salisburylaan 133, 9820 Merelbeke, Belgium; 2 Institute for Agricultural and Fisheries Research, Technology and Food Unit, Brusselsesteenweg 370, 9090 Melle, Belgium; 3 Current address: Biological Hazards (BIOHAZ) Unit, European Food Safety Authority (EFSA), Largo N. Palli 5/A, I-43121 Parma, Italy

## Abstract

Campylobacter contaminated broiler chicken meat is an important source of foodborne gastroenteritis and poses a serious health burden in industrialized countries. Broiler chickens are commonly regarded as a natural host for this zoonotic pathogen and infected birds carry a very high *C. jejuni *load in their gastrointestinal tract, especially the ceca. This eventually results in contaminated carcasses during processing. Current intervention methods fail to reduce the colonization of broiler chicks by *C. jejuni *due to an incomplete understanding on the interaction between *C. jejuni *and its avian host. Clearly, *C. jejuni* developed several survival and colonization mechanisms which are responsible for its highly adapted nature to the chicken host. But how these mechanisms interact with one another, leading to persistent, high-level cecal colonization remains largely obscure. A plethora of mutagenesis studies in the past few years resulted in the identification of several of the genes and proteins of *C. jejuni* involved in different aspects of the cellular response of this bacterium in the chicken gut. In this review, a thorough, up-to-date overview will be given of the survival mechanisms and colonization factors of *C. jejuni *identified to date. These factors may contribute to our understanding on how *C. jejuni *survival and colonization in chicks is mediated, as well as provide potential targets for effective subunit vaccine development.

## 1. Introduction

*Campylobacter *infections are now the leading cause of human bacterial gastroenteritis in many developed countries [1,2]. *Campylobacter *enteritis in humans is mainly caused by *C. jejuni *[2]. Chickens are a natural host for *Campylobacter *species and are often colonized by *C. jejuni *in particular [2]. Therefore, this review will focus on the interaction of *C. jejuni *with the broiler chick in particular. Transmission to humans probably most commonly occurs through consumption and handling of chicken meat products contaminated with this zoonotic pathogen during slaughter and carcass processing [3,4], in which *Campylobacter *colonization of the chicken intestinal tract plays an important role [5]. The chicken reservoir as a whole is estimated to be responsible for up to 80% of human campylobacteriosis cases [4]. But despite many efforts, current intervention methods fail to reduce the colonization of chickens with *Campylobacter *[6]. Intensive research in the past few years resulted in an increased insight into the colonization mechanism of *C. jejuni *in chicks, with several of its colonization factors identified. This newly gathered information, which is the topic of this review, might aid in the development of new effective vaccines to reduce *C. jejuni *prevalence in broiler flocks and eventually the number of chicken meat-related human campylobacteriosis cases.

## 2. *C. Jejuni *Colonization Pattern in Broiler Chicks

It is generally accepted that *C. jejuni *colonizes the avian gut as a commensal and colonized broilers carry a large number of bacteria in their ceca (generally around 10^6 ^to 10^8 ^cfu/g), the predominant site for colonization [7,8]. Ingestion of *C. jejuni *numbers as few as 35 cfu can be sufficient for successful colonization of chicks [9]. After ingestion, the bacterium reaches the cecum and multiplies, resulting in an established colonizing *Campylobacter *population within 24 hours after entrance [10]. Most flocks become colonized only at an age of two to four weeks [11,12], probably due to the presence of maternally-derived antibodies in young chicks conferring protection against colonization [13]. Once flock colonization is detected, the majority (> 95%) of the birds of that flock is colonized within several days [14] and stay so until slaughter [10,15].

*C. jejuni *isolates can have different colonization potential [9,16,17]. Isolates from humans have been reported to be less successful in colonizing chickens than poultry isolates [17,18]. *C. jejuni *isolates from poultry have been divided in three colonization phenotypes. Strains of the first phenotype fail to colonize 14-day-old chickens. In the second phenotype, strains can colonize but are readily eliminated and are classified as transient. The third phenotype contains strains that show efficient and sustained colonization [16,18]. These three colonization phenotypes were found to be stable and independent of in vivo passages and the amount of viable bacteria in the inoculum. Although *C. jejuni *strains did show enhanced colonization capacity (i.e. the minimal infective dose required for maximal colonization decreased) after passage through the avian gastrointestinal (GI) tract, their colonization phenotype did not change [17]. Enhanced colonization capacity and increased virulence after in vivo passage through chicks has been shown in several other studies as well [9,19,20]. This variability in colonization capacity, but the fixedness of the colonization phenotype of a given strain indicates that *C. jejuni *genes involved in initial and sustained colonization are not identical. However, in contrast to this stable colonization phenotype [17], it has been previously reported that after several in vivo passages a poorly colonizing isolate was able to consistently colonize chicks [9].

## 3. *C. Jejuni *Colonization Factors in the Chicken Gut

As in the environment, also in the chicken intestine *C. jejuni *is likely to encounter environmental stressors compromising optimal growth [21]. The persistent colonization of the chicken GI tract by *C. jejuni *indicates that the bacterium harbours regulatory systems that confer protection toward a hostile environment inside, but also outside the host. The mechanism by which the bacterium adapts to this "hostile" environment, resulting in successful and persistent colonization, is poorly understood. It is clear, however, that successful colonization of the chicken GI tract is a multifactorial process [22] in which genes involved in all areas of the colonization process of *C. jejuni *play a role. In the next sections, a thorough, up-to-date overview is given on identified colonization factors of *C. jejuni *in the chicken gut, summarized in Table 1.

**Table 1:** To date identified colonization factors of *Campylobacter jejuni *in the avian gastrointestinal tract

Functional area	Gene name or locus	Identified/predicted protein function	Reference
Multidrug efflux pump	*cmeABC* *cmeR* *cj0561c* cbrR*	*Campylobacter *multidrug efflux pump transcriptional repressor of *cmeABC* putative periplasmic protein *Campylobacter *bile resistance orphan response regulator	[24] [25] [27]
Chemotaxis	***docB*** ***docC*** *acfB* *cheY* ***tlp1*** *luxS* *cheB, cheR*	probable methyl-accepting chemotaxis protein (MCP) probable MCP protein probable MCP protein chemotaxis regulatory protein chemoreceptor transducer-like protein signal autoinducer AI-2 biosynthesis enzyme putative adaptation proteins	[32] [26] [32] [33] [35] [29]
Motility	*flaA* *maf5* *rpoN* *fliA* ***flgR*** *flgK* *cj1321 *- *cj1325/6*	major flagellin motility accessory factor (flagellar biosynthesis) RNA polymerase σ^54 ^subunit RNA polymerase σ^28 ^subunit response regulator possible flagellar hook associated protein flagellin *O*-linked glycosylation island	[44,45] [44] [32,48] [32,43] [32,48] [52]
Capsule formation and *N*-linked glycosylation	*kpsM* *pglH* *cj1496c*	high molecular weight glycan probable glycosyltransferase glycoprotein with unknown function	[44] [32,44,68] [66]
Two-component regulatory systems	***racR*** - *racS* *dccR *- *dccS* *cbrR* *cprR *- *cprS* *flgR *- *flgS*	reduced ability to colonize regulatory system diminished capacity to colonize regulatory system *Campylobacter *bile resistance orphan response regulator *Campylobacter *planktonic growth regulation regulatory system flagellar signal transduction system	[71] [72] [27] [73] [32,43]
Temperature regulation and heat shock response	*dnaJ* *racR*	heat-shock protein reduced ability to colonize response regulator	[55,75] [71]
Adhesion	*capA*? *cadF* *pldA* ***peb1A*** *flpA*	autotransporter lipoprotein outer membrane fibronectin-binding protein outer membrane phospholipase A periplasmic ABC transporter of amino acids fibronectin-like protein A	[[76] vs [77]] [55,80] [55,82] [77,116]
Invasion	*ciaB* *docB* *docC* *tlp1*	*Campylobacter *invasion antigen B probable MCP protein probable MCP protein chemoreceptor transducer-like protein	[55] [31,32] [31,33]
Iron regulation	*feoB* *fur* *cfrA* *ceuE* *cfrB* *chuA* *cj0178* *znuA*	specific transporter protein ferric uptake regulator (recessive) ferric enterobactin (FeEnt) receptor FeEnt-uptake periplasmic binding protein (dominant) FeEnt receptor hemin uptake outer membrane protein putative transferrin-bound iron utilization outer membrane receptor periplasmic component of a putative zinc ABC transport system	[89] [88] [90] [26] [88] [91]
Oxidative and nitrosative stress response	*docA* *cj0358* *sodB* *perR* *ahpC* *katA* ***ggt*** *ppk1* *ppk2* ***tatC*** *cj0379c*	putative cytochrome *c *peroxidase putative cytochrome *c *peroxidase superoxide dismutase peroxide-sensing regulon alkyl-hydroxyperoxidase peroxide stress regulon catalase *γ*-glutamyl transpeptidase polyphosphate kinase 1 polyphosphate kinase 2 twin-arginine translocase (TAT) secretion system TAT translocated molybdo-enzyme	[32,94] [26] [95] [99] [101] [102] [108] [107]
Central intermediary and energy metabolism	*frdABC *operon *aspA* *hydABCD *operon *fdhABCD *operon *oorDABC* (12) *nuo genes* *nrfA* *napAGHBLD *operon *ccoNOQP *operon *sdaA* *cj0415* *livJ* *cj0903c* *peb1A* *ggt*	fumarate reductase aspartate ammonia-lyase hydrogenase formate dehydrogenase 2-oxoglutarate:acceptor oxidoreductase NADH:ubiquinone oxidoreductase (complex I) nitrite reductase nitrate reductase *cbb_3_*-type cytochrome *c *oxidoreductase L-serine dehydratase putative oxidoreductase subunit putative amino acid ABC transporter periplasmic-binding protein putative amino acid transport protein periplasmic ABC transporter of amino acids *γ*-glutamyl transpeptidase	[109] [26,115] [110] [111] [26,114] [112] [32] [77,110,116] [99,100]

* Underlined and bold: genes having (probable) multiple functions

### 3.1. Multidrug and bile resistance

The *Campylobacter *multidrug efflux pump (CME) plays an important role in multidrug resistance in *C. jejuni*, mediating resistance to heavy metals and a broad range of antibiotics and other antimicrobial agents [23]. It is also responsible for resistance to bile salts in the chicken intestinal tract and is therefore essential for successful intestinal colonization in chickens [24]. CME is encoded by the operon *cmeABC *and consists of a periplasmic protein (CmeA), an inner membrane efflux transporter (CmeB) and an outer membrane protein (CmeC). Expression of *cmeABC *in *C. jejuni *is modulated by CmeR, functioning as a transcriptional repressor [23]. In a *cmeR *mutant, one gene in particular was upregulated most compared to the wild type strain: *cj0561c*, encoding a putative periplasmic protein [25]. It is suggested that CmeR directly inhibits the transcription of this gene. The expression of both *cmeABC *and *cj0561c *is strongly induced by bile compounds present in the chicken intestinal tract and expression of Cj0561c is increased over four-fold during chicken colonization [25,26]. Inactivation of *cj0561c *and loss-of-function mutation of CmeR resulted in reduced fitness of *C. jejuni *in chickens and impaired ability to colonize chicks, respectively [25]. Finally, a mutant in the *Campylobacter *bile resistance regulator (*cbrR*) gene, coding for the response regulator CbrR, was shown to be sensitive to bile components in vitro [27]. In addition, this mutant had reduced colonization ability in chicks indicating that also in vivo CbrR modulates resistance to bile salts in *C. jejuni*. Together these observations indicate that bile salts and multidrug resistance is crucial for *C. jejuni *to survive in the chicken gut.

### 3.2. Chemotaxis

Since *C. jejuni *is a highly motile bacterium, chemotaxis might be an important factor promoting its migration toward favourable conditions, and thus its survival in and colonization of the intestinal mucosa. For successful chemotaxis, an intact gradient-sensing mechanism, in which adaptation has a crucial role, is indispensable. The *C. jejuni *genome contains genes encoding putative adaptation proteins: a methylesterase CheB and a methyltransferase CheR, which are both involved in a methylation-dependent chemotaxis pathway [28]. A ΔcheBR mutant was shown to have a reduced ability to colonize the chick cecum [29]. *C. jejuni *is attracted by the glycoprotein mucin, the principal constituent of mucus, and also by the bile and mucin constituent L-fucose. The amino acids aspartate, cysteine, serine and glutamate, and the salts of the organic acids citrate, fumarate, α-ketoglutarate, malate, pyruvate and succinate also act as chemoattractants [30]. Additionally, L-asparagine, formate and D-lactate were recently identified as attractants of *C. jejuni *[31]. Surprisingly, in this study, *C. jejuni *was not attracted to citrate and L-fucose. All these chemicals are sensed by the transmembrane methyl-accepting chemotaxis proteins (MCP) of *C. jejuni *[31]. Hendrixson & DiRita [32] identified 22 *C. jejuni *genes involved in colonization of the chicken GI tract. Severely affected colonization capacity particularly resulted from mutation in the determinant of chick colonization gene B (*docB*), encoding a putative MCP and alternatively called chemoreceptor transducer-like protein10 (Tlp10). DocC (Tlp4), another MCP, was important for obtaining wild type colonization levels. Finally, also Tlp1 is important for chick colonization since a *tlp1*-isogenic mutant showed reduced colonization ability [32,33]. Surprisingly, all three chemoreceptors (*tlp1, tlp4 *and *tlp10*) have been identified as being important for invasion (see further) of *C. jejuni *in chicken embryo intestinal cells (used as a model for in vivo invasion in chicken gut epithelial cells), but not for chemotaxis [31]. While it is clear that these factors contribute to in vivo colonization, their precise role in colonization requires further study. The putative accessory colonization factor (*acfB*), encoding a probable MCP protein, is highly upregulated in the chick cecum and although not important in the early stages of colonization, it cannot be ruled out that it might be involved in the persistence of *C. jejuni *in the chick cecum in the presence of a developed gut flora [26]. A number of other genes as well have been associated with *C. jejuni *chemotaxis, including the *Campylobacter *energy taxis response genes *cetA *and *cetB *[34] and the chemotaxis regulatory gene *cheY*, which codes for a response regulator controlling flagellar rotation and is involved in the same signal transduction pathway as *CheBR *[28]. A *cheY *mutant was affected in its colonization potential of the chick cecum [32]. Also the production of the signal autoinducer AI-2 has been shown to be important for colonization [35]. Inactivation of *luxS*, the gene encoding the AI-2 biosynthesis enzyme, lead to a decrease in chemotaxis toward organic acids, in vitro adherence to chicken hepatoma (LMH) cells and chick colonization. These observations indicate that energy taxis may be an important force in environmental navigation by *C. jejuni*, driving the organism toward optimal chemical conditions for colonization.

### 3.3. Flagella and motility

Intact and motile flagella are important colonization factors for *C. jejuni *in chickens [36]. *C. jejuni *contains one or two polar flagella. The flagellar filament consists of multimers of the protein flagellin and is attached by the hook protein to a basal structure, embedded in the cell membrane and serving as a motor for rotation. The flagellin locus contains two adjacent genes, *flaA *(encoding the major flagellin) and *flaB *(encoding a minor flagellin). Both genes are independently transcribed, with the *flaA *gene regulated by a σ^28 ^promoter and the *flaB *gene by a σ^54 ^promoter [32,37,38]. Environmental and chemotactic stimuli modulate *flaA *and *flaB *promoter activity. Medium pH, growth temperature and the concentration of certain inorganic nutrients affect *flaB *promoter activity [39]. Lower pH, bovine bile, deoxycholate, L-fucose, high osmolarity and chemotactic effectors such as aspartate, glutamate, citrate, fumarate, α-ketoglutarate and succinate all upregulate the *flaA *promoter. Proline, high viscosity and milk fermented by *Bifidobacterium *or *Lactobacillus *strains downregulate the *flaA *promoter [40,41]. The *flaA *gene seems to be highly conserved among *Campylobacter *isolates and transcription is usually higher than that of *flaB *[42]. Transcription of σ^54^-dependent genes, necessary for assembly of the hook-basal body filament structure, is regulated by a two-component system composed of the sensor kinase FlgS and the response regulator FlgR [43]. Experiments with mutants have shown that *flaA *but not *flaB *is essential for colonization of chickens [44,45] although probably both are needed for full motility [46]. Colonization is also impaired with the mutant for the motility accessory factor 5 (*maf5*) gene, important for the formation of flagella [44,47]. Once *C. jejuni *reaches the cecum, it seems that mutants in the flagellar biosynthesis genes *rpoN *(encoding σ^54^) and *fliA *(encoding σ^28^) and the response regulator gene *flgR *could establish colonization at a high inoculation dose, albeit bacterial numbers were much lower compared to the controls and the number of chicks colonized by these mutants was extremely low [32,43,48]. Chickens exposed to the *flgR *mutants showed a delayed colonization. Moreover, a re-infection of *Campylobacter*-negative chickens was not observed. Since bird-to-bird transmission in flocks is generally considered to be very rapid, this indicates that the FlgS/FlgR system is mainly required for initial colonization and less for survival and persistence in the cecum of chicks [43]. Also the *flgK *mutant, expressing only the hook, showed diminished motility and was completely attenuated for colonizing the chick cecum [48]. Further supporting indications that flagella are important colonization factors for *C. jejuni *in chickens was given by Hiett et al. [49]. These authors demonstrated differential expression patterns of flagella proteins between a poor and a robust colonizer strain in poultry. These differentially expressed genes, coding for proteins involved in the modification of the flagellum, are located in hypervariable regions of the *C. jejuni *genome. This variability was shown to be extendable to the protein level, and thus may contribute to the survival of *C. jejuni *in its different environments and hosts.

In *C. jejuni *chicken isolates, the flagellin *O*-linked glycosylation island, responsible for successful flagellin assembly and motility, is very diverse [50]. Five genes (*cj1321 *- *cj1325/6*) lying in this variable region are, however, significantly prevalent among *C. jejuni *strains associated with poultry [51] and might therefore be important for the ability of certain *C. jejuni *strains to colonize this host. Mutagenesis and functional and structural data supported this hypothesis, with particularly *cj1324 *being important for chick colonization [52].

The flagellar apparatus functions as a type III secretion apparatus for the *Campylobacter *invasion antigens (Cia proteins) [53], important for in vitro cell invasion [54] and chick colonization [55], and secretion is enhanced upon exposure to chicken mucus [56]. A correlation has been demonstrated between chicken colonization potential and in vitro secretion of Cia proteins [56]. *RpoN *mutants are completely aflagellated and as such do not secrete Cia proteins, nor do *flgK *mutants [48], making it clear that the molecular basis behind the colonization mechanism in chickens is complex.

The role of motility of *C. jejuni *colonization in the chicken GI tract is not fully understood. Non-motile *C. jejuni *mutants can colonize chickens, be it at substantially reduced levels and only when chickens are inoculated with high amounts of viable cells [43]. Probably, motility is needed for *C. jejuni *to pass the GI tract so it can reach its protective niche, the mucus layer of the cecal crypts [7], and to resist gut peristalsis [32], hence it is important for initial colonization. It is, however, not known if motility is important in the persistence of *C. jejuni *in the intestinal tract, leading to long-term colonization. In any case it is clear that the specialized flagellum of *C. jejuni *serves multiple functions in the adaptation of *C. jejuni *to the chicken GI tract.

### 3.4. Surface-accessible carbohydrate structures and immune evasion

Several surface-accessible carbohydrate structures (SACS) such as flagella, lipooligosaccharides (LOS), a capsule and *O*- and *N*-linked glycans contribute to *C. jejuni *colonization in chicks.

In *C. jejuni*, the lipopolysaccharide molecule only consists of lipid A and the (inner and outer) core oligosaccharide and is therefore referred to as LOS, as the high-molecular-weight *O*-polysaccharide is a capsular polysaccharide not linked to the lipopolysaccharide molecule [57]. *C. jejuni *LOS is important for immune evasion in humans as well as host cell adhesion and invasion, and sialylation of the LOS outer core further enhances epithelial cell invasion [58]. Moreover, sialylated LOS results in reduced immunogenicity [59] and increased invasion potential in Caco-2 cells [60]. The majority of strains from human and chicken origin belonging to the clonal complex CC-21, an ecologically diverse and the largest complex in the general population structure of *C. jejuni*, were found to belong to one sialylated LOS class in particular, LOS class C, correlating with a high invasive potential [60]. Thus, sialylation of the LOS outer core is likely to contribute to successful colonization of *C. jejuni *in a suitable host. Genes responsible for the formation of the polysaccharide capsule, surrounding the surface of *C. jejuni *cells and possibly involved in survival, adherence and evasion of the host's immune system [57,61], also play a role in colonization of the chicken intestine by *C. jejuni*. Mutation in the capsular polysaccharide transporter gene M (*kpsM*), which results in the loss of a high molecular weight glycan, and thus absence of a capsule, abolished colonization of chickens [44,62]. A *C. jejuni *mutant for the *kpsE *gene, which is unable to express any capsular polysaccharide, was not hampered in its ability to colonize the chicken intestinal tract but the number of bacteria recovered from cecum and colon were lower compared to the control [63]. Interpretation of these results is hampered by the use of different chicken in vivo models and bacterial strains. Capsule formation and LOS biosynthesis genes are located in hypervariable regions in the *C. jejuni *genome [64], resulting in an enormous antigenic diversity among isolates.

*C. jejuni *is unique in being the only known prokaryote having an *N*-linked protein modification system, which is encoded by the *pgl *multigene locus [65,66]. The *N*-linked glycosylation pathway is responsible for post-translational modification of multiple proteins, including flagellin, and is conserved among *C. jejuni *isolates [50,67]. In contrast, the only known proteins to be modified by *O*-linked glycosylation in *C. jejuni *(see above) are flagellar subunits [50]. In humans, most of the *N*-linked glycosylated proteins are highly immunogenic with their glycosyl moieties being immunodominant while only limited antibody is generated against the protein fraction [67]. This indicates that glycosylation might offer *C. jejuni *a unique system of immune evasion by masking primary amino acid sequences. A mutant in the *N*-linked general protein glycosylation pathway gene H (*pglH*) possessed an intact capsule, but was unable to glycosylate proteins and was severely reduced in its ability to colonize the chicken intestinal tract [44,68]. Also strains with other mutations in the *pgl *locus were affected in their ability to colonize chicks [32], indicating that *N*-linked glycosylation in *C. jejuni *is an important colonization determinant. However, glycan modification of Cj1496c, a glycoprotein important for in vitro cell invasion in human epithelial cells and initial chick colonization does not seem to influence its function [66]. Moreover, most *N*-glycosylated proteins, including Cj1496c, are annotated to be periplasmatic and do not come in direct contact with host factors and the exact mechanism by which this glycosylation system contributes to colonization remains to be elucidated [66,69].

To conclude, several SACS of *C. jejuni*, including the unique *N*-linked glycans, contribute to successful colonization in chicks. Not only by mediating adhesion (see further), but also by creating an enormous antigenic diversity in *C. jejuni *isolates resulting in persistent high-level gut colonization of certain strains.

### 3.5. Two-component regulatory systems

*C. jejuni*, like all prokaryotes, responds to environmental changes by using two-component regulatory systems (TCRSs) consisting of response (R) regulators and sensor (S) kinases regulating *C. jejuni *gene expression [26,70]. A histidine kinase senses specific environmental triggers through autophosphorylation of the histidine residue. Subsequent transfer of the phosphate group to the corresponding response regulator turns it into an active transcription factor that can stimulate the differential expression of target genes, allowing *C. jejuni *to immediately respond to changing environmental conditions within the chicken gut such as several stressors, nutrients and temperature [70].

To date, five TCRSs have been identified in *C. jejuni *to be important for optimal chick colonization: FlgRS [43] and the orphan response regulator CbrR [28] (see above), the reduced ability to colonize (RacRS) system [71], diminished capacity to colonize (DccRS) [72] and *Campylobacter *planktonic growth regulation (CprRS) [73]. RacRS is responsive to temperature, and mutation of *racR *reduces the colonization potential of *C. jejuni *[26,71] (see also below). DccRS controls the expression of several genes encoding probable membrane-associated proteins [26]. Finally, CprRS is thought to control essential biological processes, stress tolerance and biofilm formation, making it possible for *C. jejuni *to adapt to different environments [73]. A *ΔcprS *mutant was reported to display a dramatic dose-dependent defect for chick colonization. Thus, it is clear that the genome of *C. jejuni *harbours multiple TCRS genes, involved in all aspects of *C. jejuni *biology, which are vital for its efficient adaptation to the chicken host.

### 3.6. Temperature regulation and heat shock response

The elevated body temperature of the chicken (42°C) as compared to humans implies the transcription of many different proteins uniquely transcribed in response to the chicken GI tract. The RacR/RacS signal transduction system responds to temperature changes and might play an important role in chicken colonization by *C. jejuni *[71]. Comparative analysis of the protein profile of wild type *C. jejuni *and *racR *mutants, revealed 11 proteins to belong to the RacR regulon. Three proteins were sequenced and were identified as RacR and two isoforms of a cytochrome *c *peroxidase homologue. A comparative study by Zhang et al. [74] revealed 15 to 20 proteins differentially expressed by at least two-fold when *C. jejuni *was grown at 37°C or at 42°C. All identified differentially expressed proteins are periplasmic proteins or major antigens of *C. jejuni*, or are involved in the metabolism or regulatory system. These proteins might play a role in adaptation to and pathogenicity in the different hosts of *C. jejuni*. DnaJ belongs to a family of heat shock proteins and plays a role in *C. jejuni *thermotolerance [75]. The *dnaJ *gene is located adjacent to *racR *and likely to be under the transcriptional control of RacR [71]. Mutation of *dnaJ *severely reduced colonization in chicks [55,75].

### 3.7. Adhesion

*Campylobacter *adhesion to epithelial cells of the chicken GI tract is believed to be an important step in successful colonization. Several studies contributed to the importance of intact flagella and adhesins, surface-exposed proteins, in chicken colonization. Mutation of the *Campylobacter *adhesion protein A (*capA*) gene, encoding an autotransporter lipoprotein, resulted in reduced capacity to adhere to human and chicken intestinal epithelial cells, reduced invasion capacity in human epithelial cells and abolished colonization in a chick model [76,77]. In another study, however, mutation of *capA *did not result in reduced colonization capacity [77]. Moreover, since this gene is absent in many *C. jejuni *poultry isolates, the genuine contribution of *capA *to successful chick colonization is unclear [77,78]. The *Campylobacter *adhesion to fibronectin (CadF) outer membrane protein was shown to bind to fibronectin, a glycoprotein of the extracellular matrix of the GI tract [79], and to be important for full binding capacity of *C. jejuni *to chicken epithelial cells [77]. Ziprin et al. [55,80] demonstrated that mutants in the genes *cadF *and *pldA*, the structural gene for phospholipase A, are impaired in their ability to colonize the cecum, indicating that these genes may play a prominent role in successful colonization. But in contrast to the highly prevalent *cadF *gene, many *C. jejuni *isolates lack the *pldA *gene [81]. Moreover, the biological function of *pldA *is not known. But due to its outer membrane localization it might be involved in maintaining the functional integrity of surface exposed adhesins in some strains [82]. Hiett et al. [49] demonstrated differential expression patterns for major outer membrane proteins in poultry between a poor and a robust colonizer strain. These differentially expressed genes are located in hypervariable regions of the *C. jejuni *genome and may contribute to the survival of *C. jejuni *in its different environments and hosts. Recently, a new adhesin, fibronectin-like protein A (FlpA), has been identified to be important for full binding capacity to chicken epithelial cells and successful colonization [77]. Konkel et al. [83] found that different *C. jejuni *strains compete for colonization in broilers and hypothesized that this is due to the sharing of common adhesins among these isolates and limited host epithelial cell binding places. This finding supports the hypothesis that adhesion is a key step in the colonization process of *C. jejuni *in chicks.

### 3.8. Invasion

Invasion might be an important colonization determinant of *C. jejuni *in chicks because mutations in *ciaB *as well as in the MCP genes *tlp1, tlp4 *and *tlp10*, important for in vitro invasion in mammalian and chicken cells respectively (see above), severely impair cecal colonization [31,55]. Studies with isolated primary intestinal cells from chickens indeed showed that *C. jejuni *was able to invade chicken cells [84,85], an unexpected feature since *C. jejuni *does not associate with chicken crypt epithelium in vivo [85]. Invasion capacity was largely strain-dependent, but overall no difference was observed between isolates from poultry or human origin. Microtubule- as well as microfilament-dependent invasion was reported, which is in accordance with results obtained from invasion experiments in human epithelial cell lines [86]. Many studies on the genes which are thought to play a role during invasion have been conducted on human epithelial cell lines, but thus far experiments on chicken primary epithelial cecal cells are lacking. While it is tempting to assume that invasion mechanisms in these cells are analogous to those in human cell lines, some differences do exist: *C. jejuni *can survive in vitro in human T84 epithelial cells by avoiding fusion with lysosomes [87], but intracellular survival seems not to be the case in the primary chicken enterocytes [84]. The lack of an immortalized chicken intestinal cell line and the complicated handling of primary chicken cecal cells clearly hamper investigation toward invasion (and other) mechanisms in chicken cecal cells. Nevertheless, the recent obtained in vitro and in vivo results described under this section suggest that invasion of *C. jejuni *in gut epithelial cells might be an important colonization determinant in vivo.

### 3.9. Iron transport and regulation

Regulation of the intracellular iron concentration is an important factor to secure colonization. Iron is essential for electron transfer processes and functions as a cofactor for several enzymes. It is also responsible for the generation of hydroxyl radicals. Moreover, iron availability modulates the transcription of genes belonging to several functional groups, thereby affecting the ability of *C. jejuni *to colonize the GI tract [88]. The soluble ferrous iron (Fe^2+^) is readily transported across the outer membrane via porins and is subsequently transported across the cytoplasmic membrane by a specific transporter protein, FeoB. This transporter is important for iron acquisition and intracellular survival of *C. jejuni*, as well as for successful gut colonization [89]. Mutants in the ferric uptake regulator (*fur*) gene, the *cfrA *gene responsible for an outer membrane ferric enterobactin (FeEnt) receptor and the *ceuE *gene encoding a FeEnt periplasmic binding protein regulated by *fur*, are all compromized in their ability to colonize chickens, with complete absence of live bacteria for the latter two [88], as were mutants in another recently identified and characterized outer membrane FeEnt receptor CfrB, which is most prevalent in *C. coli *strains [90]. Inactivation of *cfrB *in a *cfrA*-negative *C. jejuni *strain fully abolished its ability to utilize FeEnt as a sole iron source for growth. Moreover, the reduced colonization phenotype of the isogenic *cfrB *mutant of *C. jejuni *could not be restored by the presence of a functional *cfrA *gene. In contrast, complementation of an isogenic *cfrA *mutant with the wild type *cfrB *gene in *trans *fully restored the ability of this *C. jejuni *mutant to utilize FeEnt. Thus, CfrB plays an important role during colonization of *Campylobacter *in chicks and cannot be compensated by other iron uptake mechanisms without affecting the colonization potential. Therefore, it is believed that CfrB is the dominant receptor both in FeEnt utilization by and during colonization of chickens with *C. jejuni *strains producing both a functional CfrA and CfrB. Transcription levels of *chuA*, a gene believed to code for an outer membrane receptor for hemin and hemoglobin, are increased over 40-fold in the chicken cecum, indicating that ChuA might be required for *C. jejuni *to colonize chicks [26]. Finally, mutation in Cj0178, a putative transferrin-bound iron utilization outer membrane receptor, resulted in reduced colonization potential [88]. Given this information, it can be concluded that several iron-uptake systems are essential for the survival of *C. jejuni *and for its successful colonization in the chicken host.

Besides iron, also zinc has been reported to be an important trace element necessary for *C. jejuni *growth inside the chicken host [91]. A *C. jejuni *mutant lacking ZnuA, the periplasmic component of a putative zinc ATP-binding cassette (ABC) transport system, had a growth defect in zinc-limiting media and was severely affected in its colonization potential in chickens.

### 3.10. Oxidative and nitrosative stress defence

*C. jejuni *is a microaerophilic microorganism and thus requires reduced oxygen levels for its growth. Nevertheless, it must resist oxidative stress it may encounter both in the environment and in its host, like the superoxide anion, hydrogen peroxide and biotoxic hydroxyl radicals. These stressors can result from incomplete reduction of oxygen by *C. jejuni*, or be induced by the chick immune system [92]. *C. jejuni *contains a wide range of enzymes involved in defence against oxidative stress. Several of these regulators have already been identified. However, the mechanism of gene regulation in *C. jejuni *is still poorly understood. Cytochrome *c *peroxidases (CcPs) are generally responsible for the conversion of hydrogen peroxide to water [92]. In a study by Ahmed et al. [93] 23 DNA sequences, including cytochrome oxidase III, were found to be present in a robust but absent from a poor colonizer *C. jejuni *strain. No direct link could be found that these factors correlate with the identified genes by Hendrixson and DiRita [32], but it can be assumed that also these strain-specific genes are factors important for efficient and sustained colonization. *C. jejuni *has two CcP loci, which surprisingly do not contribute to hydrogen peroxide resistance and thus do not protect against oxidative stress. Instead, it seems that in *C. jejuni *resistance to hydrogen peroxide is mainly mediated by the sole cytoplasmic catalase KatA, breaking it down to water and oxygen [92,94]. Nevertheless, mutation in one of the two CcP loci, *docA*, located immediately upstream of *docB*, resulted in a substantial dose-dependant decrease in colonization potential [32,94]. Moreover, Woodall et al. [26] found Cj0358, another putative CcP, to be upregulated 12-fold in vivo suggesting a role for this protein in hydrogen peroxide removal from the periplasm. By constructing an isogenic Δ*perR *mutant, deficient in the regulon of the peroxide-sensing regulator (PerR), and comparing its transcriptome profile with that of the wild type strain, Palyada et al. [95] identified over 100 genes to be part of the PerR regulon. Mutation of *perR *significantly reduced *C. jejuni *motility and attenuated colonization in chickens. This study also revealed a functional network between the key players of the oxidative stress defence system, including mainly the antioxidant proteins encoded by the superoxide dismutase (*sodB*), defending *C. jejuni *against the superoxide anion, the alkyl-hydroperoxide reductase (*ahpC*) and *katA*, their transcriptional regulators *fur *and *perR *and the regulatory pathways that connect them. This indicates that there is a link between oxidative stress (PerR regulated) and iron metabolism (Fur regulated) in *C. jejuni *and that oxidative stress defence mechanisms and their proper regulation are essential for successful and efficient colonization of the chick cecum. Indeed, the colonization potential in chicks was reduced by 50 000-fold in the *C. jejuni ΔahpC *mutant, while in *ΔperRΔfur, ΔkatA *and *ΔsodB *mutants colonization was completely abolished. This indicates that all key players of this functional network need to be intact for successful colonization of *C. jejuni *in chicks. Garenaux et al. [96] demonstrated that next to SodB, CadF and FlaA also a periplasmic protein (Cj1371) and a two-component regulator (Cj0355c) were overexpressed following exposure to paraquat, a strong oxidizing agent. These findings suggest that both proteins play a role in *C. jejuni *oxidative stress resistance and might be important for persistent chick colonization, but this has yet to be demonstrated.

The enzyme *γ*-glutamyl transpeptidase (GGT) is involved in maintaining cellular glutathione levels. Glutathione is an antioxidant molecule providing vital cellular protection against reactive oxygen species, generated by aerobic respiration [97,98]. GGT was shown to be present in a robust but absent from a poor colonizer *C. jejuni *strain [93], suggesting that GGT activity is not needed for initial colonization but indispensable for persistence of *C. jejuni *in the avian gut [99]. GGT catalyzes the conversion of gluthatione and glutamine to glutamate, and the ability of certain *C. jejuni *strains to utilize glutamine or glutathione as a sole carbon source is absolutely dependent on the presence of GGT [100]. GGT is not present in all *C. jejuni *strains [99] which could explain the lower colonization capacity of strains lacking a functional GGT.

A *ppk1 *and *ppk2 *mutant, defective in respectively polyphosphate kinase 1 (PPK1) and 2 (PPK2), two key enzymes of the polyphosphate metabolization, were shown to have decreased invasion ability in human intestinal epithelial cells and a dose-dependent colonization defect in chicken ceca [101,102]. This indicates that the utilization and accumulation of polyphosphate helps *C. jejuni *to adapt to the cecal environment of the chick.

For survival and optimal colonization in the chick, *C. jejuni *must also be capable of eliciting a suitable response to cytotoxic nitric oxide (NO), a free radical produced by several cells of the host immune system that is bactericidal against *C. jejuni *[103]. *C. jejuni *is protected against NO-induced nitrosative stress by NO-detoxifying mechanisms, including a nitrite reductase and its single-domain *Campylobacter *globin (Cgb) [104,105]. Expression of Cgb in response to NO is not regulated by Fur nor PerR, but mediated by the transcription factor NssR, regulating a nitrosative stress-response regulon that also includes a truncated haemoglobin (Ctb) probably involved in oxygen metabolism [98,106]. NO detoxification in *C. jejuni *is believed to proceed via a Cgb-catalyzed dioxygenase or denitrosylase reaction, converting NO and oxygen to nitrate [103].

Many *C. jejuni *redox proteins essential for electron transfer (see further) have *N*-terminal twin-arginine translocase (TAT) signal sequences ensuring proper transport across the cytoplasmic membrane [107]. The TAT secretion system has been shown to be important for *C. jejuni *to cope with stress and for chick colonization [108]. A *C. jejuni tatC *knockout mutant had defects in biofilm formation, motility and flagellation, and was defective in survival under osmotic shock and oxidative and nutrient stresses, impairing the efficient transmission of *C. jejuni *to a susceptible host. The *ΔtatC *mutant was unable to persistently colonize chickens which is likely the result of multiple, additive effects caused by the inability of the *tatC *mutant to translocate essential TAT substrates [108]. Also a *cj0379c *mutant, lacking a functional TAT translocated molybdo-enzyme of unknown function, was deficient in chick colonization [107]. The nitrosative stress phenotype of this mutant suggests a role for Cj0379 in the reduction of reactive nitrogen species in the periplasm.

It is clear that within its chicken host *C. jejuni *can encounter several stressors which it must resist for successful colonization. The evidence above indicates that *C. jejuni *developed some interplaying survival mechanisms that allow the organism to cope with chicken gut-induced oxidative and nitrosative stress.

### 3.11. Central intermediary and energy metabolism

In *C. jejuni*, all enzymes necessary for a complete oxidative tricarboxylic acid cycle are present. A key step in this cycle is the oxidation of succinate to fumarate. Until recently, it was believed that in *C. jejuni *this reaction is exerted by both a fumarate reductase (Frd) and a succinate dehydrogenase (Sdh) since both enzymes were found to contribute to the total fumarate reductase of *C. jejuni *in vitro and were significantly upregulated in the chick cecum [26,109]. A *C. jejuni *mutant missing the intact FrdA subunit of the FrdABC enzyme was completely deficient in its succinate dehydrogenase activity in vitro and had reduced colonization ability in chicks. In contrast, experiments with the *sdhA *mutant of *C. jejuni *showed that Sdh exhibits no succinate dehydrogenase activity and is not required for colonization, indicating that the *sdh *operon has been misannotated. Thus, Frd is the sole succinate dehydrogenase of *C. jejuni *and is therefore essential for full host colonization [109].

To meet all of its energy demands, *C. jejuni *utilizes oxidative phosphorylation [110]. In the chicken cecum, however, *C. jejuni *encounters an environment with reduced oxygen levels to which it must elicit a suitable response to efficiently and persistently colonize this part of the gut. Microarray analysis revealed several genes involved in this response to be upregulated when *C. jejuni *enters its host compared to in vitro culture [26], with three genes in particular: the anaerobic C_4_-dicarboxylate transporter genes *dcuA *and *dcuB *as well as the aspartase gene *aspA*. Probably these genes play an important role during chick colonization. A double mutant in hydrogenase (Hyd) and formate dehydrogenase (Fdh) and a mutant in 2-oxoglutarate:acceptor oxidoreductase (OoR), had markedly reduced colonization ability in chicks, indicating the importance of these electron donor enzymes [110]. The same authors also identified NADH:ubiquinone oxidoreductase (complex I) to play an important role because a mutant in this gene showed impaired colonization capacitiy. Mutants in the respiratory enzymes nitrate reductase, nitrite reductase and *cbb_3_*-type oxidase all colonize the chicken cecum to a lesser extent [111]. Moreover, these enzymes are upregulated in the chick cecum, indicating that *C. jejuni *might utilize nitrite and nitrate, as well as fumarate as a terminal electron acceptor instead of oxygen [26]. Especially nitrate is considered as a potential in vivo electron acceptor [104]. It is suggested that the ability of *C. jejuni *to use gluconate as an electron donor is important for full colonization potential in the avian host [112]. A *cj0415 *mutant, lacking gluconate dehydrogenase (GADH) activity, was impaired in establishing colonization in chicks but not in mice, which can probably explained by the higher expression level of *cj0415 *at 42°C compared to 37°C [112].

*C. jejuni *is an asaccharolytic bacterium and is therefore entirely dependent on a tight set of amino acids including L-aspartate, L-glutamate, L-proline and L-serine and Kreb cycle intermediates as a primary carbon and energy source [113]. Mutants of the L-serine dehydratase gene *sdaA *were defective to catabolize L-serine and their colonization potential in chicks was abolished [114]. Moreover, *sdaA *was upregulated more than two-fold in *C. jejuni *upon colonizing the chick cecum, indicating the importance of serine for in vivo survival [26]. Also *aspA *has been demonstrated to be upregulated (by 4.8-fold) in the chick cecum [115]. An *aspA *mutant, which was unable to use any amino acid besides L-serine, was shown to have impaired ability to persist in the intestines of outbred chickens, which can possibly be explained by the reduced growth potential of this mutant in the avian gut, because aspartate enhances oxygen-limited growth of *C. jejuni *in an AspA-dependent way. Also mutation in one of two genes probably involved in amino acid transportation in *C. jejuni, livJ *and *cj0903c*, resulted in a marked colonization defect in chicks [32].

Due to an observed reduction in adhesion to and invasion in cultured epithelial cells the PEB1a protein has been regarded as a putative adhesin [77,116]. A *peb1A *mutant was not capable of colonizing chicks but did, however, not show a reduced binding capacity to chicken LMH cells [77]. This suggests that PEB1a serves a role other than, or next to, mediating adhesion during in vivo colonization. Indeed, the protein is mainly located in the periplasm and is believed to function as an ABC transporter of aspartate and glutamate, essential for the utilization of these amino acids as a carbon source during microaerobic growth [77,102,116]. However, the two-component signal peptide of PEB1a might be responsible for its localization both in the periplasm as on the cell surface, where it could act as an adhesin [116]. It is unclear whether PEB1a is present in the outer membrane, but it can definitely be found in the supernatant of *C. jejuni *cultures, indicating that the protein can be exported across the outer membrane. Nevertheless, no direct evidence is available that PEB1a functions as an adhesin in *C. jejuni*. Therefore, the inability of the *peb1A *mutant to colonize chicks [77] is probably attributable to the inability of this mutant to utilize glutathione, glutamine and the dipeptide *γ*-glutamylcysteine, although GGT activity is not affected. Thus, GGT allows the utilization of these nutrients by generating glutamate, which is then taken up by the PEB1a-dependent transporter and subsequently used as a carbon source [100].

To conclude, due to its assaccharolytic and microaerobic nature, *C. jejuni *is dependent on amino acids and electron acceptors other than oxygen as primary energy sources for optimal growth. Although the underlying mechanisms are not yet fully characterized, several of the key molecules and genes of the central intermediary and energy metabolism have been identified to date. It is clear that disturbance in the proper metabolism of these nutrients is accompanied by a severely hampered survival potential and colonization ability in chicks.

## 4. Vaccine Application Versus Immune Evasion

Although generally accepted that *C. jejuni *colonizes its avian host as a commensal, *C. jejuni *inefficiently adheres to and invades cells of the chicken gut epithelium [117]. This is initially followed by an inefficient inate immune response by the chick resulting eventually in the production of specific antibodies [117,118]. Although such a response is not able to clear *C. jejuni *from the gut, reduced bacterial counts have been observed [118]. Moreover, if antibodies against *C. jejuni *are already present in the chick the ability of *C. jejuni *to colonize is dramatically reduced, be it due to transfer of maternal antibodies to or through immunization of such birds [13,118]. Therefore, the recent identification of many of the factors needed by *C. jejuni *to colonize the chicken gut opens the way for subunit vaccine development to eradicate this pathogen from poultry flocks.

Potential vaccine candidates must be expressed during and being important for colonization in chicks. In addition they should ideally be highly immunogenic, conserved and prevalent among *C. jejuni *isolates. Although some promising results were obtained focussing on *C. jejuni *outer membrane proteins (OmpH1 and Omp18) and FlaA as subunit vaccine candidates, no effective commercial vaccine against *Campylobacter *in chicks is available to date [6].

Bacterial OMPs are regarded as promising vaccine components because of their accessibility for the host immune system and the key roles they play in the host-bacterium cross-talk [119]. CadF and CfrA may therefore hold much promise for such applications. Not only are they highly conserved and prevalent in *C. jejuni *strains, but these surface-exposed proteins are also highly immunogenic in chicks [118,119]. Moreover, antibodies directed to CfrA were recently suggested to hinder the interaction of FeEnt with this receptor [91,119]. *C. jejuni *periplasmic PEB1 can possibly be transported across the outer membrane and is highly immunogenic in humans. Whether this immunogenicity can be extended to the chicken host is not known, but clearly PEB1 deserves further attention as a possible candidate for vaccination studies in chicks. Finally, Cj0178, the secreted CiaB and transmembrane Tlp-10 may be immunogenic but their precise role during chick colonization has yet to be determined.

Also *C. jejuni *surface-exposed polysaccharide structures may be promising candidates for subunit vaccines. Indeed, several genes essential for successful colonization (*kpsM, flaA, flgK, pglH, maf5 *and *cj1324*) are involved in SACS biosynthesis. However, most SACS are highly variable and implicated in immune evasion in humans. Whether this can be extended to the chicken host is not clear. In any case, identification of conserved polysachharide epitopes of SACS is critical for exploiting these structures for vaccine application.

Finally, a plethora of other *C. jejuni *factors are indispensable for chicken gut colonization. These include *znuA, cj0379, docA, docB, perR, fur, ceuE, katA, dnaJ *and *sodB*, as well as the (highly) conserved *cj0415, tatC *and *ppk1 *genes. Their gene products are, however, not known to be surface-expressed, but rather reside in the peri- or cytoplasm where they exert their vital roles. As a consequence, they do not come in direct contact with the chick immune system. Nevertheless, their identification significantly contributes to a better understanding in the *C. jejuni *biology during chick colonization. Therefore, it could be useful to examine whether (and how) also these targets could be exploited for *C. jejuni *control in poultry.

## 5. Concluding Remarks

Poultry is a natural host for zoonotic *Campylobacter *species and the broiler chicken gut is often colonized by *C. jejuni *in particular. As a result, chicken meat products are considered to be the main source of campylobacteriosis in humans. Despite many efforts, no effective strategy exists to clear this pathogen from chickens, in part due to the poor understanding of their dual interaction. Besides genes which are probably necessary during colonization of the GI tract in a wide range of animal species in general, it seems that *C. jejuni *needs a distinct set of gene products for optimal adaptation to the unique aspects of the chicken intestinal environment, resulting in high-level cecal colonization. And although information about genes important for *C. jejuni *colonization in chicks is increasing and some cooperative functional networks (e.g. iron metabolism/oxidative stress defence) crucial for colonization are starting to unravel, the mechanisms by which these factors interplay to form the basis behind the complex interaction of *C. jejuni *with its avian host remain largely unclear. Nevertheless, we can now conclude that several factors and processes, involved in all branches and stages of the *C. jejuni *cellular response, are crucial for the adaptation of this bacterium to the chicken gut and thus indispensable for the organism to colonize its avian host. Some of these critical colonization determinants may be exploited by researchers in the field to develop new, effective vaccines to eradicate this zoonotic pathogen from poultry flocks. Especially CadF, CfrA, Tlp-10, CiaB and PEB1 seem promising targets and further research, including identification of their functional and conserved epitopes, could result in the identification of factors capable of targeting a wide range of the circulating *C. jejuni *strains in poultry.

In conclusion, intensive research in the last few years resulted in the identification of several of the chicken colonization determinants of *C. jejuni*. Further research must give a better insight of how these factors interplay, forming the functional network that is responsible for the highly adapted nature of this organism to the avian gut. Unravelling these mechanisms might aid in the development of more efficient control measures for clearing this zoonotic pathogen from poultry lines, thereby reducing the number of human campylobacteriosis cases associated with consumption and handling of contaminated poultry meat products.

## Competing interests

The authors declare that they have no competing interests.

## Author's information

The views and findings in this article are solely those of Dr Winy Messens and do not necessarily reflect the views or position of the European Food Safety Authority.

## Authors' contributions

DH drafted the manuscript. KVD and FP helped to draft the manuscript. AM, FVI, WM, MH and FH were involved in revising the manuscript critically for important intellectual content. All authors read and approved the final manuscript.
